# Identification of Factors Influencing Cholesterol Changes in Elementary-School Children: A Longitudinal Study

**DOI:** 10.3390/children9040518

**Published:** 2022-04-06

**Authors:** Hiromi Kawasaki, Satoko Yamasaki, Hazuki Shintaku, Susumu Fukita

**Affiliations:** 1 Graduate School of Biomedical and Health Sciences, Hiroshima University, Hiroshima 734-8551, Japan; morisato@hiroshima-u.ac.jp (S.Y.); pekeponsukopun@outlook.jp (H.S.); 2 School of Nursing, Dokkyo Medical University, Tochigi 321-0293, Japan; s-fukita@dokkyomed.ac.jp

**Keywords:** cholesterol, lifestyle factors, school health, cardiovascular health

## Abstract

Changes in serum cholesterol levels during childhood may affect the risk of cardiovascular disease in adulthood. However, cholesterol level changes in Japanese children and adolescents and the factors that influence them have not been completely elucidated. This study aimed to determine whether cholesterol levels changed due to the effects of growth and identify factors. This cohort study included elementary-school children in the fourth grade (9–10-year-old) who underwent assessments of cholesterol levels and demographic and lifestyle characteristics. The participants were followed up in their first year of junior high school with the same laboratory, demographic, and lifestyle assessments. From the fourth year of elementary school to the first year of junior high school, children’s cholesterol levels decreased (*p* < 0.0001). Regarding bowel movements, cholesterol level reduction was significant in individuals with regular bowel movements but not significant in those with infrequent bowel movements. Weight was the factor that most strongly negatively predicted cholesterol level reduction (*p* < 0.001). The study demonstrated the significance of lifestyle factors for growth-related changes of total cholesterol levels and identified weight as the factor that most strongly influenced total cholesterol level changes. Guidance regarding lifestyle improvements should be imparted to children from the fourth grade of elementary school.

## 1. Introduction

The high prevalence of dyslipidemia in children is a serious problem in modern society [1]; a previous study conducted on a cohort of 9–10-year-old Japanese children showed that 15% of the children had increased total cholesterol levels [2]. The historical trends of changes in cholesterol levels in children and adolescents have been investigated, but the results vary depending on the study [3,4,5,6,7].

Many of the hypercholesterolemia cases in children represent familial hypercholesterolemia [8]. Notably, cholesterol levels in children change with age and may decrease due to growth. Therefore, the risk of hypercholesterolemia is often considered to be low in children. However, the changes in cholesterol levels in community-living healthy children have not been definitively elucidated.

In modern society, the lifestyle of children has started to resemble that of adults, with reduced physical activity, a lack of exercise, and altered nutritional habits [9,10]. Such significant changes pose crucial challenges regarding the risk of developing hyperlipidemia, obesity, and other lifestyle-associated medical conditions. Thus, childhood obesity has increasingly become a concern [11,12]. Moreover, a sedentary lifestyle and unhealthy nutrition may increase cholesterol levels. In addition, because of the lockdown situation due to the COVID-19 pandemic, the use of electronic devices, particularly tablets and smartphones, has increased [13]. Isolation and the prolonged sitting and use of electronic devices for long periods have increased the prevalence of sedentary habits and lifestyles in children. In light of these developments, the Japanese Ministry of Education, Culture, Sports, Science and Technology has emphasized the need for the resumption of sports [14]. Furthermore, one previous study noted an association between cholesterol levels and nutritional knowledge as well as behaviors in children and their guardians [2]. Another study by Scherr and colleagues iterated the significance of lifestyle factors for cholesterol levels in children [15]. 

Childhood hypercholesterolemia has been associated with long-term health risks. Thus, LDL-cholesterol levels in children were correlated with the intima-media thickness of the common carotid artery, a preclinical marker of atherosclerosis, in adulthood [16]. It has been reported that cholesterol screening in childhood may facilitate the preventive management of cardiovascular disorders in adulthood [17]. However, the early prevention of hypercholesterinemia in children has not been emphasized. Since it is difficult to test healthy children and to evaluate the effectiveness of health guidance, the active engagement of children attending school in prophylactic activities has been impeded [18]. Children should be motivated to participate in hypercholesterolemia-preventing activities that would help them establish favorable lifestyle habits by adulthood. Moreover, the characteristics of younger children who need assistance with establishing healthy lifestyle habits should be clarified, which would allow for the prioritizing of health guidance provisions in schools without performing blood tests [19].

This study aimed to determine whether cholesterol levels change with growth and to clarify the situation related to their change in order to enable the development of materials for the health guidance of 9–10-year-old elementary school students. 

## 2. Materials and Methods

### 2.1. Study Participants

Children of the applicable grade, who lived in Akitakata City, attended public schools, and were 9–10 years old, were recruited in compliance with the mayor’s policy that emphasized disease prevention. The mayor was concerned regarding the prevalence of lifestyle-related diseases among young people, which would put pressure on medical expenses. Another concern regarding Akitakata City was its declining population. Akitakata City is a mountain city with population of 27,000 people and a population density of 50.1 people/km^2^, which is much lower than that of Hiroshima City (1318 people/km^2^). The declining population will disrupt the mutual aid system for medical expenses.

**Figure 1:**
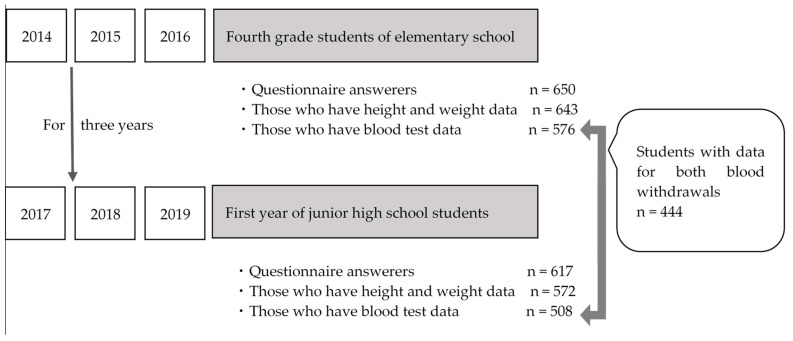
A flowchart presenting the recruitment procedure.

The participating children and their parents gave consent for the examination. The Municipal Corporation of Akitakata City agreed to the data analysis. In all three years of primary assessments (2014, 2015, and 2016), the children assessed were 9–10 years old, and were all in the same grade (fourth grade of elementary school). Children who were in the fourth grade of elementary school in 2014, 2015, and 2016, would have turned 10 years old during the periods 2 April 2014–1 April 2015; 2 April 2015–1 April 2016; and 2 April 2016–1 April 2017, respectively. They were 3 years older during the second assessments in 2017, 2018, and 2019, respectively. The recruitment procedure is presented in Figure 1.

### 2.2. Study Survey

The same survey was conducted in the fourth grade of elementary school and first year of junior high school. The survey was developed as a collaboration among schoolteachers, health care nurses, and our team. It was based on the Japanese Ministry of Education, Culture, Sports, Science and Technology’s survey of children. The questionnaires were filled out by the children themselves at school and were collected by teachers. The data entry was completed by a contracted company, and its correctness was verified. The variables analyzed in the questionnaire are presented in Table 1.

### 2.3. Data Overview

Blood tests were limited to the laboratory analyses included in the general health checkups for adults due to cost considerations. For blood testing, 10–15 mL blood was withdrawn using venipuncture. The blood samples were handled in accordance with the general health examination procedures in Japan and sent by the school for analysis to a company contracted by the municipality. The participants underwent blood tests twice: in the fourth year of elementary school and first year of junior high school. 

A guide was mailed to the schools from the city’s health center. The guide described the method for blood withdrawal and preparation for it as well as the details of the analysis. It reminded children to check whether they had an allergy to rubbing alcohol. Moreover, it indicated that the cost of the test would be paid in full by the local government, and that the data obtained would be used by the school health teachers to develop guidelines for the children. Further, it indicated that detailed analysis would be conducted by Hiroshima University. Finally, it also advised that the parents and children should be explicitly asked whether they consented to the procedure. The guide also advised that, as a precaution, if a child refused the test on the planned day, it should not be conducted. The study information was also distributed to the parents by the local government followed by registration for blood sampling of the children performed by their parents. The children watched an educational video on blood collection prior to the procedure. For cholesterol analysis, total cholesterol was assessed using the cholesterol oxidase method. In Japan, total cholesterol levels are categorized as follows: acceptable <190 mg/dL, borderline 190−219 mg/dL, and high >220 mg/dL [20]. The school physician holistically determined the need for treatment based on the reference data for 10-year-old and for 13-year-old children published by the National Center for Child Health and Development.

Weight, height, and BMI values were also included in the study. Japanese children are required to undergo height and weight measurements at least once a year before June. The data were maintained by school health teachers.

### 2.4. Statistical Analysis

The statistical analysis was performed using the data of participants whose data from both the surveys and blood tests were available for both evaluation timepoints. The need for guidance due to cholesterol changes according to the children’s ages and the association of increased cholesterol levels with their appearance and habits were analyzed using two-sample and paired t-tests. The association between changes in the cholesterol level and growth related to height, weight, and body mass index (BMI) was analyzed by a Pearson correlation analysis, and the Pearson correlation coefficient was defined as *r.* Multiple regression analysis was also conducted to evaluate lifestyle habits due to the difference between two cholesterol levels with the change in cholesterol levels as the objective variable and parameters measured in the fourth grade of elementary school as explanatory variables. Finally, we analyzed whether appearance and habits determine the need for health guidance without a blood test. The STATA statistical software was used for statistical analysis. *p*-values < 0.05 were considered statistically significant.

## 3. Results

### 3.1. Demographic Characteristics of the Study Participants

The demographic characteristics of the participants in the study, who were in the fourth grade of elementary school, are presented in Table 1. The study population included similar numbers of male and female students. The majority of the participants had bowel movements every day but not at a fixed time (50.5%); 49.9% of the participants were satisfied with their body shape, and 67.5% of the individuals included in the study had never contemplated losing weight. Moreover, the amount of time spent on games, TV, the internet, smartphones, or phones each day after returning from school had large variations. The majority of participants (61.5%) did not know about lifestyle-related diseases. The height, weight, BMI values, BMI z-scores, and height z-scores of the participants in the fourth grade of elementary school and first year of junior high school are presented in Table 2.

### 3.2. Longitudinal Cholesterol Level Changes and Their Relationship with Demographic and Lifestyle Characteristics

The mean cholesterol level in the fourth grade of elementary school was 174 mg/dL (SD 24.8 mg/dL). It decreased to 166 mg/dL in the first year of junior high school (SD 24.7 mg/dL) (Table 3). A statistically significant difference was found between the cholesterol values at the two measurement timepoints (*p* < 0.0001) using *t*-test.

Longitudinal changes in serum cholesterol levels and their association with the demographic and lifestyle characteristics of the participating children were analyzed using two-sample t-tests (Table 4). The total cholesterol levels significantly decreased from the fourth grade of primary school until the first year of junior high school (*p* < 0.0001). The reduction in total cholesterol levels was significant in both boys (*p* < 0.0001) and girls (*p* = 0.0299). Regarding bowel movements, the reduction in cholesterol levels was significant in individuals who had bowel movements every day but not in those who had bowel movements less often. Interestingly, cholesterol levels decreased in participants who wanted to lose weight or thought that their body shape should be as it is, but not in individuals who wanted to gain weight. Children who spent at least a little time but less than 3 h per day on a video game, computer, television, or phone showed a decrease in cholesterol levels. Particularly, a reduction in cholesterol levels was observed in individuals who considered themselves healthy sometimes or always. Interestingly, a cholesterol level reduction was detected in individuals who could not explain lifestyle-related diseases at the beginning of the study.

### 3.3. Correlation of Cholesterol Levels and Changes with Growth- and Weight-Related Factors

Table 5 describes the analysis of the correlation between cholesterol levels and changes related to growth and weight with continuous variables. The weight and cholesterol level changes (*p* = 0.0002) as well as weight and cholesterol levels in the first year of junior high school (*p* = 0.0402) were negatively correlated. In the first year of junior high school (*p* = 0.0369), cholesterol levels and height were negatively correlated. Moreover, there were strong correlations among cholesterol levels and their changes. The correlation between total cholesterol levels in the fourth grade of elementary school and their changes was negative, and the correlation between total cholesterol levels in the first year of junior high school and their changes was positive (Table 5).

### 3.4. Factors Predicting Changes in Cholesterol Levels Revealed by Multivariable Regression Analysis

The baseline factors predicting changes in cholesterol levels revealed by a multivariable regression analysis are presented in Table 6. Weight was the factor that most strongly negatively predicted the reduction in cholesterol levels (*p* < 0.001), meaning that in individuals with greater weight, the cholesterol levels were less likely to be reduced. There was a positive predictive effect of height (*p* = 0.041), indicating a higher likelihood of cholesterol level reduction in taller individuals.

## 4. Discussion

There is an urgent need to understand how cholesterol levels change in children and adolescents and to elucidate the factors influencing these changes. Our survey and analysis showed that cholesterol levels decrease in children from the fourth year of primary school until the first year of junior high school. In particular, weight was the most consistent factor associated with cholesterol levels; lower weight was a predictor of a significant cholesterol level reduction. The findings of this study support the need for teachers and public health authorities to instruct children whose cholesterol level changes are unpredictable.

The changes in lifestyle in modern society that also affect children have caused concerns regarding the incidence of dyslipidemia in the pediatric population. Studies from different geographic areas demonstrated an increased prevalence of high cholesterol levels among children [2,21,22]. Moreover, the risk of dyslipidemia in children is a strong argument for the implementation of cholesterol screening [23]. Nutrient–gene interactions should also be considered to fully understand the complexity of cholesterol level regulation [24]. 

Previous studies indicated an association between lifestyle factors and cholesterol levels in children, suggesting the significance of nutritional knowledge, behavior, and physical activity for the maintenance of cholesterol levels within the reference range [2,12,25,26,27,28]. Moreover, an association between obesity and dyslipidemia has been observed in pediatric populations [22]. Furthermore, the age at which the BMI increases in children is apparently also important for tackling the risk of metabolic syndrome [29]. During adolescence, fat distribution is affected, and adiponectin reportedly decreases. Adiponectin concentration was shown to play an important role in lipid metabolism [30]. Moreover, low adiponectin levels were observed in adolescents with a high weight [31]. 

The presence of multiple risk factors such as obesity, decreased physical activity levels, and unhealthy dietary habits in children has been correlated with a particularly high atherogenic index [32]. Our findings confirm the important role of weight in cholesterol levels using a prospective design, which enabled us to follow the longitudinal development of cholesterol levels. However, previous studies reported that even young people with normal weight may be at risk for dyslipidemia [33]. Furthermore, height should be considered when analyzing the cholesterol levels of pubertal children [34]; this idea was also confirmed by our findings. However, our study found no significant association between cholesterol changes and BMI values. The average 50th percentile BMI values for each age in Japan [35] and the BMI values of the subjects in the current study were roughly the same. In other words, children in the target area apparently had BMI values within the average range for Japan.

There are still uncertainties regarding the health guidance for overweight children aged 9–10 years. Our findings also show that children with a heavy weight cannot expect a significant decrease in cholesterol levels simply due to growth, but cholesterol levels may be expected to decrease with growth and good habits; thus, children should be provided with specific longitudinal guidance. Accordingly, our results highlight the need to guide children aged 9–10 years regarding health-promoting lifestyle habits. Previous studies indicated that obesity prevention programs may improve the health outcomes in school children [36]. The current investigation suggests that an obesity prevention program intended for younger children may be valuable for improving cholesterol levels, thereby decreasing the risk of cardiovascular disease in adulthood. Although the effect is recognized, the interests of children of this age and their parents are directed towards their educational studies. This is because elementary school students and parents are less aware of the importance of lifestyle-related diseases, which rarely occur in this age group [37]. Reportedly, in pre-adolescent children, obesity and health risk do not correlate [38]. This risk is believed to be induced by diet and lifestyle until adolescence [30]. The findings of our study support the idea that cholesterol lowering is pronounced in children with good lifestyle habits. This knowledge contributes to the motivation for elementary-school students to develop habits that prevent lifestyle-related diseases. Our study suggests the need of health guidance considering weight and lifestyle in the fourth grade of elementary school. 

Limitations of the study include the relatively low number of laboratory assessments and the fact that self-report measures, which may not realistically represent the children’s habits, were used in some cases. Moreover, the regulation of cholesterol levels and the correlation with different factors that influence them may be different at high levels versus normal levels or in obese children versus normal weight children. Finally, the number of participants in some subcategories was low, and the findings should be replicated in larger studies. Future studies should also design and examine the effect of specific lifestyle modifications on the longitudinal cholesterol changes in youth.

## 5. Conclusions

Our data indicate the significance of weight and lifestyle factors in longitudinal changes in total cholesterol levels in school-aged children. They also show that that children with a heavy weight cannot expect a significant decrease in cholesterol levels simply due to growth, but cholesterol levels may be expected to decrease with growth and good habits. This knowledge is useful for motivating elementary-school students to adopt strategies for the prevention of lifestyle-related diseases. In addition, our findings clearly suggest that lifestyle-related guidance is required from the fourth grade of elementary school.

## Figures and Tables

**Table 1:** Demographic characteristics of the study participants when they were in the fourth grade of elementary school.

Variables	n	%
Gender		
Female	321	49.4%
Male	329	50.6%
Total	650	
Do you have a bowel movement every day?		
May not have a bowel movement for 3 days or more	41	6.3%
May not have a bowel movement for 1–2 days	198	30.4%
I have a bowel movement every day but not at fixed times	328	50.5%
I have a bowel movement every day at a fixed time	74	11.4%
Unknown	9	1.4%
Do you know your weight?		
I do not know it	240	36.9%
I know it	397	61.1%
Unknown	13	2.0%
Please tell me how you feel regarding your body shape		
I want to be quite thin	72	11.1%
I want to be a little thinner	192	29.5%
It should be as it is	324	49.9%
I want to gain a little weight	47	7.2%
I want to gain a lot of weight	6	0.9%
Unknown	9	1.4%
Have you ever tried to deliberately lose weight?		
I was told by a hospital or schoolteacher that I was overweight, and I was instructed to do it	2	0.3%
I did it because I wanted to lose weight	102	15.7%
I wanted to, but I have not done it yet	98	15.1%
Never thought about it	439	67.5%
Unknown	9	1.4%
How much time do you spend each day playing video games, on TV, the internet, smartphones/phones, etc. after returning from school?		
None	49	7.5%
Less than 1 h	182	28.0%
1 h or more and less than 2 h	164	25.2%
2 h or more and less than 3 h	115	17.7%
3 h or more	131	20.2%
Unknown	9	1.4%
Do you think you are healthy?		
No	11	1.7%
Seldom	72	11.1%
Sometimes	337	51.9%
Yes	220	33.8%
Unknown	10	1.5%
Can you consciously improve your mood?		
No	62	9.5%
Seldom	118	18.2%
Sometimes	209	32.1%
Yes	254	39.1%
Unknown	7	1.1%
Do you know about lifestyle-related disease?		
No	400	61.6%
A little	171	26.3%
I cannot explain them, but I think I know	60	9.2%
I can explain them	7	1.1%
Unknown	12	1.8%

**Table 2:** Height, weight, and BMI values of the participants.

Variable	N	Mean	Standard Deviation
Height in the fourth grade of elementary school	643	132.7	6.13
Weight in the fourth grade of elementary school	643	30.3	6.15
BMI in the fourth grade of elementary school	643	17.1	2.55
BMI z-score in the fourth grade of elementary school	643	3.15	3.32
Height z-score in the fourth grade of elementary school	643	0.03	1.00
Height in the first year of junior high school	572	152.2	8.35
Weight in the first year of junior high school	572	44.6	9.33
BMI in the first year of junior high school	572	19.4	6.88
BMI z-score in the first year of junior high school	572	0.88	3.49
Height z-score in the first year of junior high school	572	0.29	1.23

**Table 3:** Cholesterol levels in the analyzed sample.

Variable	N	Mean	Standard Deviation
Total cholesterol (mg/dL) in the fourth grade of elementary school	444	174	24.8
Total cholesterol (mg/dL) in the first year of junior high school	444	166	24.7
Average total cholesterol change (mg/dL)	444	−8.1	20.7

Note: A statistically significant difference was found between the cholesterol values at the two measurement timepoints (*p* < 0.0001) using paired *t*-test.

**Table 4:** *t*-test comparison between mean total cholesterol levels at two time points.

Variable	Fourth Grade of Elementary School	First Grade of Junior High School	*p*-Value
Total	142.8	127.7	<0.0001
Gender			
Female	148.3	139.0	0.0299
Male	137.6	116.6	<0.0001
Do you have a bowel movement every day?			
May not have a bowel movement for 3 days or more	147.7	127.3	0.0654
May not have a bowel movement for 1–2 days	141.5	132.1	0.0804
I have a bowel movement every day but not at fixed times	142.8	127.1	0.0011
I have a bowel movement every day at a fixed time	149.8	121.0	0.0018
Do you know your weight?			
I do not know it	137.0	124.5	0.0193
I know it	146.5	129.2	0.0001
Please tell me how you feel regarding your body shape			
I want to be quite thin	155.2	135.1	0.0235
I want to be a little thinner	143.9	125.3	0.0030
It should be as it is	142.1	128.3	0.0034
I want to gain a little weight	139.0	122.2	0.1082
I want to gain a lot of weight	108.3	131.3	0.6488
Have you ever tried to deliberately lose weight?			
I was told by a hospital or schoolteacher that I was overweight, and I was instructed to do it	140.5	143.5	0.5307
I did it because I wanted to lose weight	146.4	128.2	0.0148
I wanted to, but I have not done it yet	143.3	131.7	0.1284
Never thought about it	142.9	126.7	0.0001
How much time do you spend a day on video games, TV, the internet, smartphones/phones, etc., after returning from school?			
None	144.0	132.2	0.1638
Less than 1 h	151.2	134.8	0.0028
1 h or more and less than 2 h	146.1	129.6	0.0087
2 h or more and less than 3 h	139.5	121.4	0.0278
3 h or more	146.6	133.2	0.0725
Do you think you are healthy?			
No	136.6	138.4	0.5177
Seldom	138.6	132.1	0.2501
Sometimes	142.9	128.1	0.0021
Yes	146.0	124.8	0.0002
Can you consciously improve your mood?			
No	126.4	122.6	0.3830
Seldom	144.5	132.6	0.0725
Sometimes	145.3	127.0	0.0020
Yes	145.9	127.6	0.0006
Do you know about lifestyle habits?			
No	141.9	127.5	0.0011
A little	144.8	131.2	0.0266
I cannot explain them, but I think I know	152.1	118.8	0.0012
I can explain them	123.6	110.9	0.3759

Note: The comparisons were carried out using two-sample *t*-tests.

**Table 5:** Correlation between cholesterol levels/changes and continuous growth- and weight-related variables.

	TC Timepoint 1		TC Timepoint 2		Change in TC	
	r	*p*-Value	r	*p*-Value	r	*p*-Value
Height	−0.0599	0.2074	−0.0991	0.0369	−0.0467	0.3265
Weight	0.0477	0.3163	−0.0974	0.0402	−0.1733	0.0002
BMI	−0.0364	0.4447	−0.0638	0.1796	−0.0327	0.4914
TC Timepoint 1	1		0.6496	<0.0001	−0.4192	<0.0001
TC Timepoint 2	0.6496	<0.0001	1		0.4179	<0.0001
Change in TC	−0.4192	<0.0001	0.4179	<0.0001	1	

Abbreviations: BMI = body mass index; TC = total cholesterol; timepoint 1 = fourth grade of elementary school; timepoint 2 = first year of junior high school. Note: Pearson product moment correlation coefficient analysis was conducted; r is the product moment correlation coefficient.

**Table 6:** Factors predicting changes in cholesterol levels revealed by multivariable regression analysis.

Variable	Coefficient	*p*-Value	Low CI	High CI
Height	0.42	0.041	0.02	0.83
Weight	−0.92	<0.001	−1.34	−0.50
BMI	−0.03	0.263	−0.08	0.02

Abbreviations: BMI = body mass index; CI = confidence interval.

## Data Availability

The data are not publicly available because of confidentiality reasons.
